# Gelatin-Oxidized Alginate and Chitosan-Coated Zein Nanoparticle Hydrogel Composite to Enhance Breast Cancer Cytotoxicity in Dual-Drug Delivery

**DOI:** 10.1021/acsomega.4c06404

**Published:** 2024-10-31

**Authors:** Sanierlly da Paz Do Nascimento, Ramon Ramos Marques de Souza, Marianna Vieira Sobral, Francisco Humberto Xavier-Junior, Marcus Vinícius Santos da Silva, Marcelo Machado Viana, Fausthon Fred da Silva, Michael J. Serpe, Antonia L. de Souza

**Affiliations:** † Postgraduate Program in Chemistry, Universidade Federal da Paraíba, João Pessoa, Paraíba 58051-900, Brazil; ‡ Postgraduate Program in Natural and Synthetic Bioactive Products, Universidade Federal da Paraíba, João Pessoa, Paraíba 58051-900, Brazil; § Department of Pharmaceutical Sciences, Universidade Federal da Paraíba, João Pessoa, Paraíba 58051-900, Brazil; ∥ Institute of Physics, Federal University of Bahia, Salvador, Bahia 40170-290, Brazil; ⊥ CTNano, Universidade Federal de Minas Gerais, Belo Horizonte, Minas Gerais 31270-901, Brazil; # Department of Chemistry, Universidade Federal da Paraíba, João Pessoa, Paraíba 58051-900, Brazil; ∇ Department of Chemistry, University of Alberta, Edmonton, Alberta T6G 2R3, Canada

## Abstract

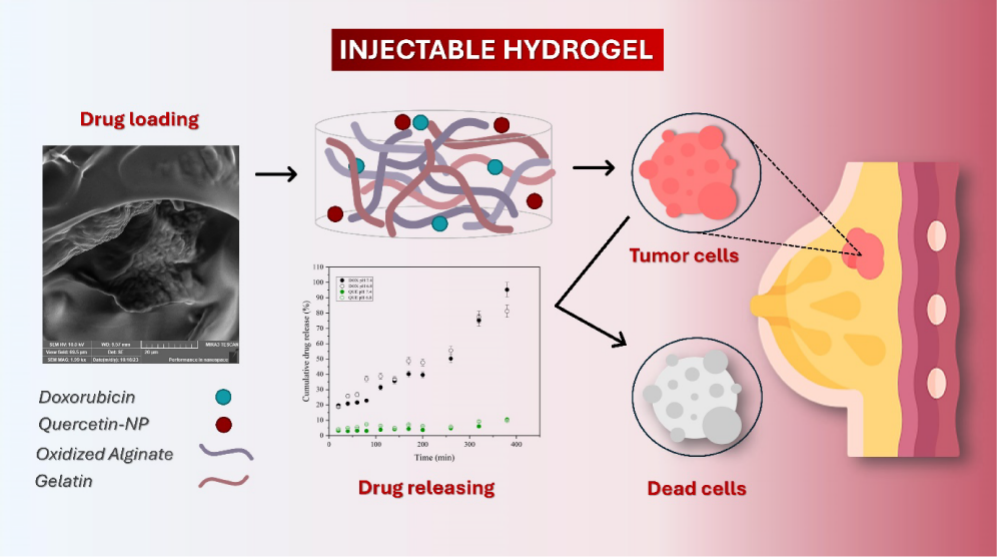

This study explores the combined delivery of doxorubicin and quercetin using a gelatin-oxidized alginate-based hydrogel as a promising strategy for localized breast cancer therapy. Our approach involves the incorporation of doxorubicin within the hydrogel matrix and loading quercetin into chitosan-coated zein nanoparticles. The hydrogel exhibited self-healing properties attributed to Schiff base cross-linking and demonstrated injectability. Characterization of its microstructural, mechanical, and textural properties revealed a porous and flexible structure, demonstrating its suitability for drug release applications. Both drugs exhibited distinct in vitro release profiles at pH 6.8 (typical of tumor tissue), with doxorubicin at 81.2% and quercetin at 9.7%. After 72 h of release, the cytotoxicity against MCF-7 breast cancer cells was assessed. The hydrogel formulation containing doxorubicin increased the cytotoxic action by 4.66-fold, whereas the hydrogel composite, containing both doxorubicin and quercetin-loaded nanoparticles, enhanced it by 20.7-fold compared with doxorubicin alone. Thus, the findings of our study highlight the enhancing effect of the dual release system, thereby expanding the utility of gelatin-oxidized alginate-based hydrogels as advanced drug delivery systems, as exemplified by the combined delivery of doxorubicin and quercetin.

## 1 Introduction

Doxorubicin (Adriamycin) is a crucial chemotherapy drug known for targeting various cancer types.^1^ However, its efficacy in inhibiting cancer cell proliferation is limited by systemic toxicity and poor tumor-specific delivery.^2,3^ Mainly considering that conventional forms of administration of chemotherapy drugs normally cause undesirable systemic side effects, leading to poor clinical treatment, affecting quality of life, and making it difficult for patients to accept them.^4^ Localized drug delivery systems offer a promising solution to enhance outcomes and reduce side effects,^3^ especially in breast cancer, where targeted methods can significantly improve treatments outcomes.

Drug delivery systems using biomaterials stood out as a true revolution in cancer treatment, with reduced side effects, making the treatment more humane for the patient as well as increasing the effectiveness of some chemotherapeutic agents. In this context, hydrogels have gained attention as drug delivery platforms for cancer therapy due to their tunable properties and biocompatibility. Recent reviews have extensively highlighted their efficacy and versatility.^5,6^ Among them, gelatin-oxidized alginate hydrogel (Schiff base) has shown exceptional potential as a polymeric matrix. This is due to its biodegradability and bioprintability.^7^

Research on oxidized alginate hydrogels has investigated properties such as reinforcement with microhydroxyapatite for bone tissue engineering applications.^8^ Other studies applying these hydrogels to wound healing treatment^9,10^ and myocardial infarction treatment^11^ were also reported. Besides that, hydrogels and nanoparticles can be combined to form nanocomposite systems for drug delivery, where hydrophobicity plays a role. For example, zein nanoparticles derived from corn grains, are characterized by their biocompatibility, biodegradability, low toxicity, and ease of preparation, used as effective delivery system to therapeutic agents.^12^

It is worth considering that this would be a strategy explored for the dual administration of drugs. However, other studies have investigated the use of oxidized alginate for drug delivery focused on individual drug release scenarios, not evaluating dual drug delivery.^13,14^ Some studies have even reported the use of drug-carrying nanoparticles as a strategy to improve therapeutic performance, such as chemotherapy drugs, which can have a better reach to the tumor, since nanoparticles, due to their small size, are more permeable to cellular structures, in addition to having a longer life in the circulatory system.^15^

Considering these aspects, some studies have shown an association between doxorubicin and quercetin, a flavonoid extracted from plants. Due its anticancer properties,^16^ combining quercetin with chemotherapeutic agents can synergistically induce tumor cell apoptosis and reduce drug resistance.^17,18^ Quercetin, in particular, enhances doxorubicin’s *in vitro* cytotoxicity^19^ and reduces cardiac toxicity from the doxorubicin-cyclophosphamide regimen.^20^ However, quercetin’s hydrophobicity limits its use in water-based systems, making nanoparticle encapsulation ideal for its delivery from hydrogels.^21^

Thus, this work incorporates doxorubicin within the hydrogel matrix and loads quercetin onto chitosan-coated zein nanoparticles, enabling the localized release of both hydrophilic and hydrophobic drugs. This study aims to produce and characterize a dual-drug delivery hydrogel for cancer therapy. We investigated its physicochemical properties, drug release kinetics, and *in vitro* cytotoxicity against cancer cells.

## 2 Experimental Section

### 2.1 Materials and Methods

Zein from corn (∼98%), sodium carbonate (Na_2_CO_3_, ∼99.8%), chitosan (50–190 kDa), sodium alginate (W201502), sodium metaperiodate (∼98%), sodium carbonate, phosphate-buffered saline solution (PBS), quercetin (QUE), and doxorubicin (DOX) were purchased from Sigma-Aldrich (Merck, Brazil). All solvents and chemicals were of analytical grade and were used as received. We acquired commercial gelatin from a local market and used it without further purification. Ultrapure water was obtained using a Milli-Q purification system. The MCF-7 cell line was obtained from the Rio de Janeiro cell bank (BCRJ, Brazil).

### 2.2 Preparation of Zein Nanoparticles

Chitosan-coated hollow zein nanoparticles (HNP) were obtained using sodium carbonate as a sacrificial template through the method reported by Xu and Khan and their collaborators^22,23^ with some modifications. The process involved combining 3.5 mL of absolute ethanol with 5.0 mL of a 50 mg/mL zein solution in a 70% ethanol–water mixture. We added to this mixture 1.5 mL of a Na_2_CO_3_ (1%) solution, and the resulting solution was stirred magnetically at 1000 rpm for 1 min. Posteriorly, the solution was added to 40 mL of ultrapure water. The nanoparticles were mixed with 1 mg/mL chitosan in a 1% acetic acid solution, using a 1:1 volume ratio, while stirring at 1000 rpm for 30 min. We removed ethanol in the particle dispersion using a rotary evaporator EV400H–V model (Labtech, Sorisole, Bergamo, Italy) at 35 °C for 30 min. Unstable particles were separated by centrifuging the dispersion at 4230 rpm for 15 min, followed by centrifuging the supernatant at 9550 rpm for 30 min to remove unabsorbed chitosan. The resulting precipitate was then dispersed in an equal volume of water to obtain HNPs. To prepare chitosan-coated quercetin-loaded zein nanoparticles (QNP), we added 10 mg of quercetin to a 50 mg/mL zein stock solution in an ethanol–water mixture, which corresponds to a final concentration of 250 μg/mL quercetin in the particle dispersion.

### 2.3 Preparation of Hydrogel Composites

Prior to hydrogel preparation, sodium alginate (SA) (5% w/v) underwent oxidation using sodium metaperiodate (theoretical oxidation degree 60 mol %), resulting in the introduction of aldehyde groups within the alginate chains, oxidized sodium alginate (OSA). The degree of oxidation achieved was 49.74% and the process of alginate oxidation was conducted according to the phase diagram outlined in a study by Emami and collaborators.^24^ For the hydrogel composite system (OSAGC), we added the required amount of QNP to gelatin to create a solution (10% w/w). Following that, an equal volume of 10 wt % oxidized alginate containing 10 mg of DOX in 0.1 M PBS and 15 wt % gelatin was mixed at 37 °C for 10 min and poured into silicon molds to complete the gelling reaction overnight. Oxidized alginate and gelatin (OSAG) hydrogels without the nanoparticle’s addition were produced following the above steps without introducing the particles. The hydrogel composite system containing the HNP was called OSAGH.

### 2.4 Characterization of Zein Nanoparticles

#### 2.4.1 Particle Size and Zeta Potential

We diluted the samples by a factor of 200 with ultrapure water and then analyzed them using a Zetasizer Nano ZS particle size analyzer (Malvern Panalytical, Malvern, Worcestershire, United Kingdom) at 25 °C. The size distribution of the particles was obtained using the NNLS function, while the zeta potential was estimated using phase analysis light scattering (PALS). Each measurement was performed in triplicate and reported as the mean value, accompanied by the standard deviation.

#### 2.4.2 Loading Capacity and Efficiency of Quercetin

The QNPs underwent centrifugation at 4230 rpm for 15 min to eliminate any unencapsulated quercetin. The resulting supernatant and samples were then diluted 50-fold in ethanol. QUE absorption was measured at a wavelength of 373 nm by using a UV2550 UV–vis spectrophotometer (Shimadzu Corporation, Tokyo, Japan). We constructed a calibration curve at concentrations ranging from 1 to 20 mg/mL, with a correlation coefficient of 0.999 (as shown in Figure S1. Each measurement was performed in triplicate. The loading efficiency and capacity of QUE in the particles were determined using the equations below. The results were reported as the mean value, accompanied by the standard deviation.
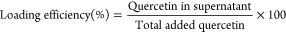
1
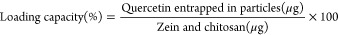
2

#### 2.4.3 Spectroscopic Characterization

After freeze-drying, QNP and HNP samples were evaluated by attenuated total reflectance Fourier transform infrared (ATR-FTIR) equipped with a Golden Gate single reflection ATR-FTIR attachment using an IRPrestige-21 FT-IR Spectrometer (Shimadzu Corporation, Tokyo, Japan). The measurements were conducted at room temperature within the range of 600–4000 cm^–1^. We obtained the infrared spectra of raw materials using the same instrument, after pressing it into a transparent pellet with KBr, within the range of 400–4000 cm^–1^, at a resolution of 4 cm^–1^, and a total of 20 scans.

#### 2.4.4 Thermogravimetric Analysis

Thermogravimetric Analysis (TGA) of HNP, QNP, and their respective raw materials was conducted in a thermogravimetric analyzer DTG-60H model (Shimadzu Corporation, Tokyo, Japan) by ramping the temperature from 30 to 400 °C at a rate of 10 °C per minute. Differential Scanning Calorimetry (DSC) analyzer model HMV-2T (Shimadzu Corporation, Tokyo, Japan) was also used to investigate the raw material and particle phase transformation process from 30 to 600 °C, nitrogen atmosphere at 50 mL/min flow rate. An additional degradation isotherm was evaluated for each particle, hydrogel, and the respective raw materials.

### 2.5 Hydrogel and Hydrogel Composite Characterization

#### 2.5.1 Spectroscopic and Thermogravimetric Analysis

After freeze-drying, OSAG hydrogel, OSAGH and OSAGC hydrogel composites, along with their respective raw materials, underwent FTIR and ^1^H NMR evaluation as previously described. ^1^H NMR spectra of hydrogel raw materials were recorded on a Bruker AM-400 spectrometer at 400 MHz, in D_2_O and DMSO. We performed thermal analysis using both TGA and DSC techniques as mentioned earlier.

#### 2.5.2 Gelling Time, Injectability and Self-Healing Properties

We conducted the gelling time determination via tube inversion method.^25^ This simple and well-known method consists of registering the time required for the mixture of the two hydrogel precursor solutions to become a gel, i.e., the time in which the gel does not flow when the tube/vial is inverted. All experiments were done in triplicate at 37 °C with tube (7.5 mm radius) inversion every 10 s. The result was reported as the mean value, accompanied by the standard deviation.

The injectability property of the OSAG hydrogel was investigated by extruding the hydrogel disc through a syringe of 2 mL with a 21-gauge needle at 37 °C.^26^ An OSAG hydrogel was prepared by using a cylindrical mold (10 mm diameter and 5 mm height) and subsequently divided into two pieces. One half of the hydrogel was left untouched, while the other was immersed in a saturated methylene blue dye solution until it was fully colored. The two opposite halves were then rejoined and incubated at 37 °C. Visual observations were made at different time points to assess self-healing and diffusion of the dye through the seam. The surface morphology of the healed hydrogel was examined by using scanning electron microscopy (SEM) after freeze-drying, as described below.

#### 2.5.3 Swelling Behavior

To investigate the swelling kinetics of the OSAG hydrogels, after their preparation, they underwent freeze-drying for 24 h and then were immersed in a pH 7.4 and 6.8 PBS solution and allowed to swell at room temperature. At specific time intervals, we removed the samples from the solution, excess water on the surface was gently wiped off, and the samples were weighed.^26,27^ The swelling ratio was then determined by using the following formula:
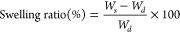
3

Here, *W*_*s*_ represents the weight of the hydrogel when it is in a swollen state at a specific time, pH, and temperature; while *W*_*d*_ represents the weight of the hydrogel when it is completely dry. The results were reported as the mean value accompanied by the standard deviation.

#### 2.5.4 *In Vitro* Degradation

The hydrogels were separately subjected to hydrolytic degradation under pH 7.4 and pH 6.8 PBS solutions at a temperature of 37 °C. Before the analysis, we lyophilized the OSAG hydrogels for 24 h. The weight loss of these cross-linked hydrogels was monitored at specific time intervals using the gravimetric technique.^28^ We performed degradability tests in triplicate, and the average values were considered. The degradation rate was determined by calculating the difference in mass loss of the hydrogels by using the following equation:
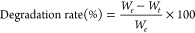
4

Where, *W*_*t*_ represents the mass of the hydrogel at a specific time, and *W*_*e*_ represents the mass of the hydrogel in the equilibrium swollen state. The results were reported as the mean value accompanied by the standard deviation.

#### 2.5.5 Rheology Measurement

We assessed the rheological characteristics of the OSAG hydrogels using a Haake Mars III rheometer (Thermo Fischer Scientific, Massachusetts, USA) equipped with parallel plate geometry (diameter: 35 mm, gap: 0.4 mm). Initially, samples were positioned on the lower plate, and strain-dependent assessments were conducted to identify the linear viscoelastic range of the hydrogels within the 0.05–10 Pa interval at a temperature of 25 °C. Subsequently, a frequency sweep analysis was executed, maintaining 1 Pa constant and examining the range from 0.05 to 10 Hz at 25 °C to ascertain the values of *G*′ and *G*″.

#### 2.5.6 Texture Analysis

Texture analysis was performed on cylindrical hydrogel samples measuring 12.5 mm in diameter and 20 mm in height using a texture analyzer TA-XT plus C (Stable Micro Systems, Surrey, UK). The method described previously^29^ was followed for the texture profile analysis (TPA), cylindrical hydrogel samples were utilized, and a P75 probe was employed (12.7 diameter). The samples were subjected to testing with a pretest speed of 2.0 mm/s, a test speed of 2.0 mm/s, a posttest speed of 2.0 mm/s, a compression distance of 5 mm, and a trigger force of 5.0 g.

#### 2.5.7 Microstructural Analysis

The morphology of the hydrogel and hydrogel containing QNPs was assessed using an MIRA3 LMH FE-SE Model (Tescan Orsay Holding, Brno, Czech Republic) operating at an accelerating voltage of 10 kV with different magnifications used. All samples were affixed to the surface of double-sided carbon tape and coated with a thin layer of gold prior to Scanning Electron Microscopy (SEM) analysis. Hollow nanoparticle (HNP) morphology analyses were determined in two different equipment using two methodologies. Transmission electron microscopy (TEM) micrographs of the chitosan-coated hollow zein nanoparticles were obtained by using a Tecnai G2–20 FEI SuperTwin 200 kV microscope. The samples were prepared by ultrasonic dispersion in isopropanol and then dropped onto a holey carbon 300 mesh copper grid. In the analysis using a JEM-2800, 200 kV microscope model (JEOL USA Inc., Peabody, USA) equipment, was accurately determined the size and shape detail of HNP. The zein nanoparticles (HNP) were deposited on a copper TEM grid coated with Formvar and carbon, using a suspension of the HNPs prepared in ultrapure water. The sample was negatively stained with 2% uranyl acetate. Additionally, atomic force microscopy (AFM) was performed using a Shimadzu SPM-9700 in phase mode (noncontact), with a super sharp probe with a curvature radius of less than 10 nm. The HNP was deposited onto freshly cleaved muscovite mica slides from a suspension prepared in ultrapure water.

#### 2.5.8 *In Vitro* Drug Release of the Composite Hydrogel

Buffer solutions with pH values of 6.8 and 7.4 were prepared and placed in a 37 °C incubator. We submerged the composite hydrogel containing 10 mg/mL of each drug in 12 mL of pre-equilibrated buffers. The incubator was set to 100 rpm at 37 °C, and at specific time intervals, a 2 mL sample of the buffer solution was removed and analyzed. The exact volume of the removed buffer was replaced to maintain the sink conditions. To prevent any evaporation-related losses, the chambers remained covered, and we exposed them only during the sampling process, which was conducted in triplicate.

To determine the drug released at different time points, concentrations were determined by a UV2550 UV–vis spectrophotometer (Shimadzu Corporation, Tokyo, Japan). Before the absorbance of the samples was recorded, autozero and standard measurements were performed to ensure accurate results. We measured the released amounts of doxorubicin at a wavelength of 478 nm, while the 373 nm wavelength was for the quercetin. We constructed calibration curves for QUE as described before, and for DOX at concentrations ranging from 1 to 40 mg/mL, with a correlation coefficient of 0.999 (as shown in Figure S2. The results were reported as the mean value accompanied by the standard deviation. We also applied the release kinetics data to five typical drug release models (zero-order, first-order, Higuchi, Korsmeyer-Peppas, and Peppas-Sahlin).

### 2.6 *In Vitro* Cytotoxicity

The cytotoxicity of the nanoparticles containing QUE (QNP), hydrogel containing DOX (OSAGX), hydrogel containing QNP (OSAGQ) and hydrogel composite (OSAGC) against the MCF-7 breast adenocarcinoma cell line was assessed using the MTT (3-(4,5-dimethylthiazol-2-yl)-2,5-diphenyltetrazolium bromide) reduction assay, which evaluates cell viability and proliferation by measuring the reducing activity of mitochondrial and cytoplasmic enzymes. The isolated drugs DOX and QUE were also examined.

The cells were seeded in 96-well plates at a concentration of 1 × 10^5^ cells/mL. Treatment solutions were prepared with a concentration of 12 μg/mL of DOX and/or QNP. These solutions were added well by well with the aid of an Eppendorf Multipette M4, in volumes of 2, 4, 6, 12, 24, 50, and 100 μL. The final volume of each well was 200 μL, completed with a culture medium. Following these parameters, the samples were added to the plates and incubated for 72 h.

After incubation, 110 μL of the supernatant was removed, and 10 μL of MTT solution (5 mg/mL) was added. Plates were incubated for an additional 4 h, after the addition of 10% sodium dodecyl sulfate hydrochloric acid solution, and the absorbance was measured using a spectrophotometer (BioTek Instruments microplate reader, Sinergy HT, Winooski, VT, USA) at a wavelength of 570 nm. Three independent experiments, each performed in triplicate or quadruplicate, were conducted, and the data are expressed as the concentration that inhibits cell growth by 50% (IC50) ± standard error of the mean, determined by nonlinear regression analysis.

## 3 Results and Discussion

### 3.1 Nanoparticle Characterization

#### 3.1.1 Particle Size and Zeta Potential

As shown in Table 1, the HNP size increased with the inclusion of 100 μg/mL of quercetin. In addition, the PDI results lower than 0.3 indicate a narrow size distribution. The ζ-potential measurement yielded positive values for both hollow and loaded-particles, the particles’ surface strong cationic nature was attributed to the presence of the outer layer of chitosan, which is a natural cationic polymer.^30^ The disparity in the magnitude of the ζ-potential value may be attributed to the adsorption of quercetin on the surfaces of the particles. Nonetheless, absolute positive ζ-potential (above 20 mV) suggests a tendency for electrostatic repulsion between particles, contributing to their stability and dispersion in aqueous solutions.^31^ Moreover, the presence of a positive charge on the particle surface also holds potential for interactions with negatively charged biological entities, which could be advantageous for layer-by-layer fabrication of nanoparticles.^32^

**Table 1:** Characteristics of HNP and QNP: Size, Polydispersity Index (PDI), and ζ-Potential^a^

Particle	Particle size (nm)	PDI	ζ-Potential (mV)
HNP	195.0 ± 0.44	0.153 ± 0.006	71.4 ± 2.95
QNP	218.8 ± 2.77	0.170 ± 0.006	52.5 ± 3.80

a Where, HNP: empty nanoparticles; QNP: nanoparticles + quercetin.

#### 3.1.2 Quercetin Encapsulation in the Nanoparticles

We assessed the particle loading capacity and efficiency to quantify the extent of quercetin incorporated into the particles. The loading capacity was determined to be 4.78 ± 0.00% of quercetin, meaning the amount of quercetin that we successfully loaded per unit weight. This loading capacity highlights the zein-particle’s capability to accommodate the quercetin payload. This result falls within the range of those of Khan and collaborators^23^ who also found loading capacity results ranging from 2.22 to 5.89% when they varied the quercetin concentration. We also found results of the same magnitude (5.6%) when quercetin was loaded to caseinate/kappa-carrageenan-coated zein particles using a similar fabrication method.^33^ The loading efficiency, calculated as 67.32 ± 0.06%, reflects the proportion of the maximum theoretical loading capacity that was achieved. This indicates an effective utilization of the zein particle’s available empty space for encapsulating the particles. This empty space is created using Na_2_CO_3_ as a sacrificial core when it is precipitated in ethanol.^22^ It is known that hollow or core–shell nanoparticles provide better encapsulation and release properties, although controlling the size of the nanoparticles is still a challenge.^34^ Finally, we could argue that the slightly lower results when compared to the literature might be due to the adaptation of the encapsulation strategy apparatus in our research, which may have led to quercetin adsorption on the particles surface.

#### 3.1.3 Spectroscopic Characterization

In Figure S3, the FTIR spectrum of HNP showed a shift of the band at 3313 cm^–1^, present in the zein FTIR spectrum, to 3309 cm^–1^ suggesting hydrogen bonding between chitosan and zein, in addition to the presence of bands at 1658 cm^–1^ and 3442 cm^–1^ related to chitosan.

Consistent with the literature, amide I and II absorption bands changed from 1660 and 1571 cm^–1^ in chitosan and 1660 and 1535 cm^–1^ in zein spectra to 1658 and 1546 cm^–1^ in chitosan-coated zein nanoparticles, due to hydrophobic and electrostatic interactions among zein and chitosan.^23,35^ From Figure S4, the characteristic bands of quercetin in 1614, 1355, 1240, and 1163 cm^–1^ attributed to the stretching vibrations of C=C, C–OH, C–O–C, and C–OH groups, respectively. They are not displayed in the HNP and QNP spectra. The absence of these bands is indicative of the entrapment of quercetin in the zein-particles, since it limits the stretching and bending of some bonds.^23^ The only common absorption bands among quercetin, HNP and QNP spectra are the ones at 3302 cm^–1^ related to the O–H stretching vibrations and the ones at 1658 cm^–1^ attributed to C=O groups.

In Figure S5, the FTIR spectrum of gelatin and oxidized alginate (OSA) exhibit characteristic absorption bands at 3421 and 3442 cm^–1^, corresponding to the stretching vibrations of OH groups, respectively. The same corresponding peak in the empty hydrogel spectrum appears shifted, which indicates hydrogen bonding between the polymeric matrix components during the formation of the hydrogel. Some interesting evidence of the hydrogel formation is the absence of the shoulder at 1732 cm^–1^ in the hydrogel spectrum, which is characteristic of CHO supporting the successful oxidation of alginate; as well as the intensification of a sharp band at 1606 cm^–1^ resulted of the shifting bands at 1641 and 1612 cm^–1^ to a lower wavelength. These findings are related to the formation of Schiff base network-forming groups (imine) from the interaction of aldehyde and amino groups present in the OSA and gelatin structures, respectively.^36^ From the spectra of Figure S6, it is important to highlight the absence of a band between 1535 and 1546 cm^–1^ characteristic of amide II (present in both hydrogels containing hollow and quercetin-loaded nanoparticles). An explanation for this might be related to its proteinaceous nature, since this band is only present as a sharp and intense peak in zein, HNP and QNP spectra, but in gelatin’s spectrum it only appears as a low-intensity peak. Once again, the characteristic peaks of quercetin do not appear on the composite hydrogel spectrum, as we expected due to its encapsulation, as previously discussed.

In terms of the hydrogel raw materials, OSA and gelatin were subjected to ^1^H NMR spectroscopy to examine their structural modification and verify the absence of impurities, respectively. As shown in Figure S7, the ^1^H NMR spectrum of oxidized alginate displayed polysaccharide characteristic peaks from δ 4.5 to 5.8 ppm related to protons of the anomeric region (*highlighted in yellow*) and the signals from the region of 3.5–4.5 ppm are consistent with protons of sugar rings (*in blue*), which are consolidated in the literature.^37^ Here, the most important aspect of this spectrum is what qualitatively characterizes and confirms the structural modification of SA, which should be the typical aldehyde signal at approximately 9.7 ppm. Instead, what we observed was the appearance of two new signals at 5.15 and 5.4 ppm (see expansions in red), which would correspond to a hemiacetal proton formed from neighboring aldehyde and hydroxyl groups.^24,38^ The finding was later confirmed by ^1^H–^13^C HSQC (400 MHz, D_2_O) spectra of both SA and OSA polysaccharides (Figure S8).

In the ^1^H NMR spectrum of gelatin (Figure S9), resonances according to the expected chemical shifts of multiple amino acid residues are consistent with its proteinaceous complex nature. The amino acid residues contributing to these peaks include proline, alanine, and glycine in the aliphatic region of the spectrum, while the amide region may contain signals from peptide bonds indicating the presence of amino acid residues, all of which are consistent and have been better detailed in the literature.^39,40^ Moreover, scarcely any impurities were discernible in the NMR spectrum of gelatin.

### 3.2 Hydrogel and Hydrogel Composite Characterization

#### 3.2.1 Hydrogel Gelling Time, Injectability and Self-Healing Properties

We produced the OSAG hydrogels by the formation of imine dynamic covalent bonds (Schiff base) reaction between OSA (−CHO) and gelatin (−NH_2_). The gelling time or gelation time was determined during the tube inversion test and occurred at 2.33 ± 0.03 min after combining the precursor solutions at 37 °C inverting the tube, and no fluidity was exhibited within a 30-s duration. The gelling time of hydrogels is a critical factor in their biomedical use. It must strike a balance; it should neither be too short, which could result in clogging the needle, nor too long, as this would prevent it from conforming to the injection site and might interfere with the drug release time.^27^ In addition, the OSAG hydrogel sol–gel phase transition behavior was assessed by rheology.

**Figure 1:**
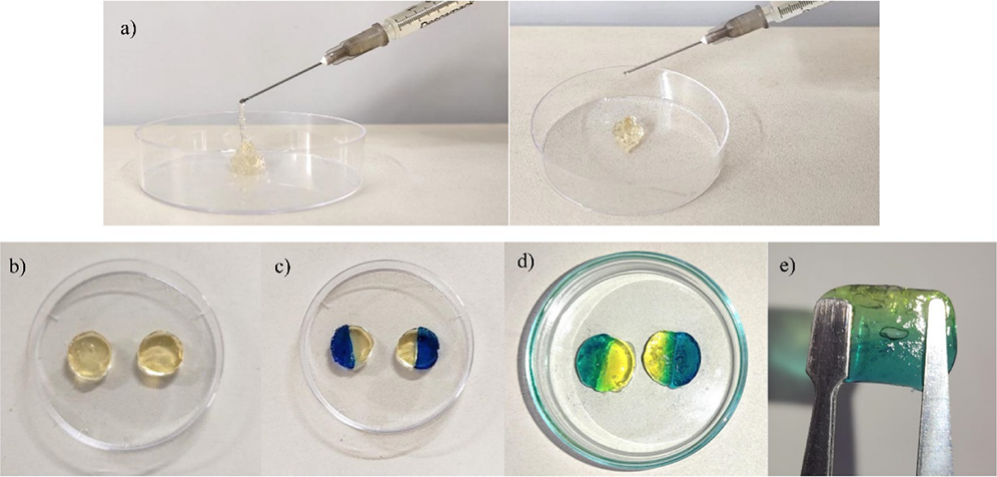
Injectable a) and extrudable behavior of preformed OSAG hydrogel, b) OSAG hydrogel disks prior to self-healing test, c) self-healing test after 1 min and d) after 24 h, and e) Self-healing capability of injected OSAG hydrogels.

The OSAG hydrogel injectability was assessed by adding both precursor solutions in the syringe and forcing it to extrude through the needle. The formed hydrogel was easily extruded through the needle, as can be seen in Figure 1a. The dynamic nature of the Schiff base covalent bond within the hydrogel network also suggests that the hydrogels present the hydrogels’ ability to self-heal. This characteristic allows for better control over the placement of hydrogels and uniform distribution of substances within living organisms.^41^

To visually examine the self-healing capability of the OSAG hydrogels, we conducted a macroscopic self-healing test. We cut two hydrogel disks (Figure 1b) in half and put each half together with the opposite one stained with distinct colors (Figure 1c). Figure 1d presents the OG hydrogel disks after one min of healing at room temperature and 24 h later, respectively. After 24 h, the self-healed hydrogel could stand independently while maintaining its integrity, as shown in Figure 1e.

#### 3.2.2 Hydrogel Swelling Behavior

**Figure 2:**
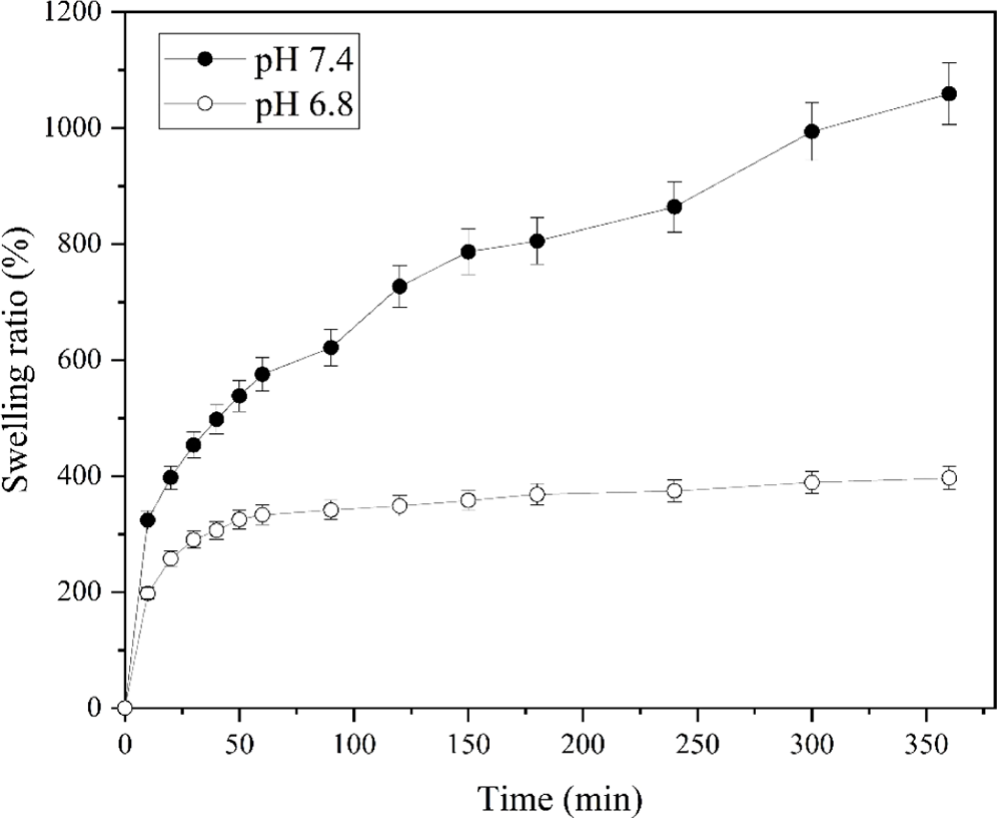
Swelling ratio of the OSAG hydrogels in pH 7.4 and 6.8 PBS buffer in the time range from 0 to 360 min at room temperature.

The swelling behavior of OSAG hydrogel was evaluated in physiological conditions (pH 7.4) and in mimicking tumor cells environment (pH 6.8).^42^ On one hand, upon immersion in a phosphate-buffered saline (PBS) solution pH 7.4, the hydrogel exhibited rapid and significant swelling within the initial hour, as can be seen in Figure 2.

The swelling ratio increased steadily throughout the analysis, reaching a maximum of 1050 times its original dry weight. We can see that its behavior reveals an initial burst phase, where rapid water absorption resulted in a quick increase weight (0–50 min), followed by a slower and more sustained swelling phase (from 50 min onward). The hydrogel reached equilibrium swelling after approximately 6 h of immersion, demonstrating its ability to retain water over an extended period.

On the other hand, upon immersion in a PBS solution pH 6.8 (mildly acidic), the hydrogel exhibited distinctive swelling behavior relevant to its potential application in cancer therapy.^42^ Notably, there was no pronounced initial burst of swelling, and the hydrogel’s expansion remained limited, not exceeding a 500-fold increase in weight. At pH 7.4, however, the hydrogel swells more due to the increased ionization of carboxyl groups in both gelatin and alginate structures, which results in higher osmotic pressure and electrostatic repulsion. This does not occur at pH 6.8, where the carboxyl groups are less ionized, leading to a lower electrostatic repulsion and reduced swelling. These findings demonstrate the hydrogel’s significant water absorption capacity and its potential for encapsulating and delivering therapeutic agents under both physiological and slightly acidic conditions, such as those found in the tumor tissue environment.

#### 3.2.3 Hydrogel *In Vitro* Degradation

**Figure 3:**
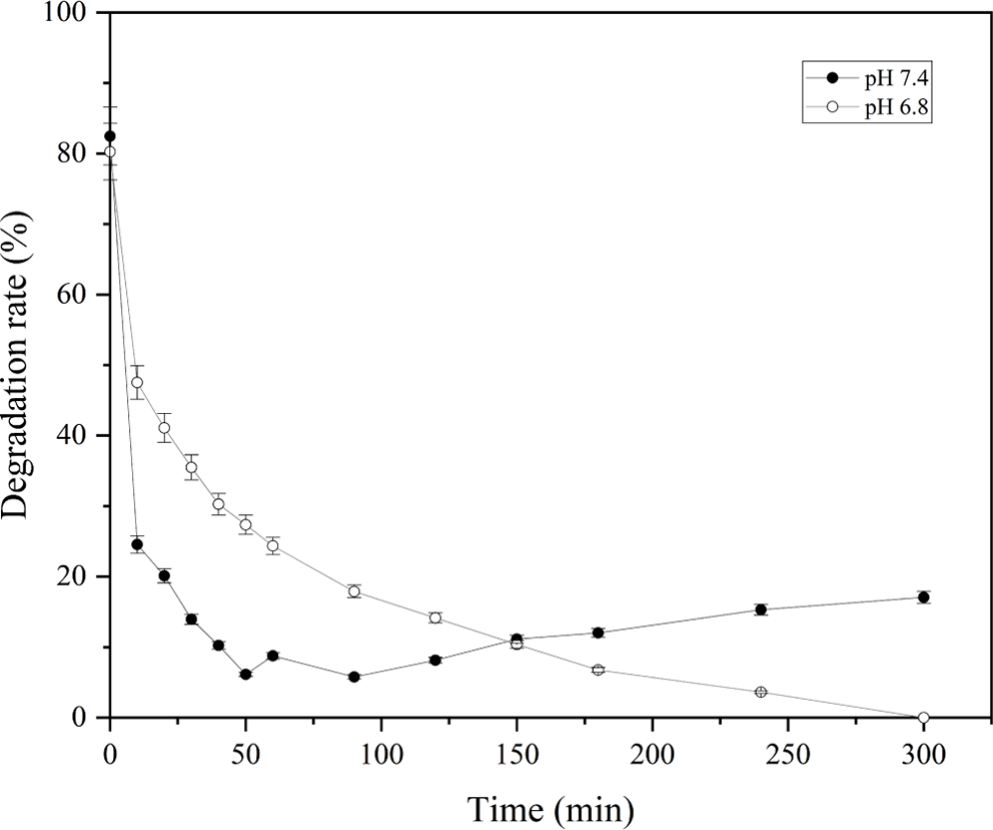
Degradation rate of the OSAG hydrogels in the time range from 0 to 300 min at 37 °C and pH values of 7.4 and 6.8.

The OSAG hydrogel stability and potential for controlled drug release was assessed under physiological conditions (pH 7.4) and a slightly acidic environment (pH 6.8) at 37 °C, as seen in Figure 3. In the first 50 min, the degradation rate is significantly higher at pH 6.8 compared to pH 7.4. Over time, the degradation rate decreased for both pH levels. At pH 6.8, the degradation rate drops rapidly and then plateaus around 20% after approximately 150 min.

Whereas at pH 7.4, the degradation rate decreases more slowly and stabilizes around 10–15%. Throughout the 300 min duration, the degradation rate remains consistently higher at pH 6.8 than at pH 7.4. Thus, this indicates that the hydrogel is more stable and degrades less at pH 7.4 compared with pH 6.8. This degradation pattern can be correlated to the previously observed swelling results performed at room temperature.

At pH 7.4, the hydrogel showed a more substantial swelling during the swelling tests. This higher expansion likely led to an alteration in pore size, with larger pores observed in the structure, due to increased ionization of carboxyl groups, which stabilizes the structure and reduces degradation.^43^

Interestingly, at pH 7.4, the degradation rate initially decreases rapidly but shows an increase at later time points. This behavior could be attributed to an adaptation phase during the initial 100 min, where the hydrogel undergoes structural adjustments in response to the elevated temperature (37 °C), differing from the conditions of swelling experiments conducted at room temperature. After this period, the hydrogel begins to swell, although not at the same rate as observed in the swelling experiments, likely due to the continued influence of the temperature on its structural dynamics. At pH 6.8, reduced swelling and ionization, associated with subsequent cross-linking hydrolysis, might have led to less stable hydrogel networks, which resulted in faster degradation.

Finally, the previous swelling properties at different pH values played a crucial role in the subsequent degradation kinetics, which suggests that hydrogel expansion and structural changes during swelling can influence and likely govern degradation rates.

#### 3.2.4 Thermogravimetric Analysis

**Figure 4:**
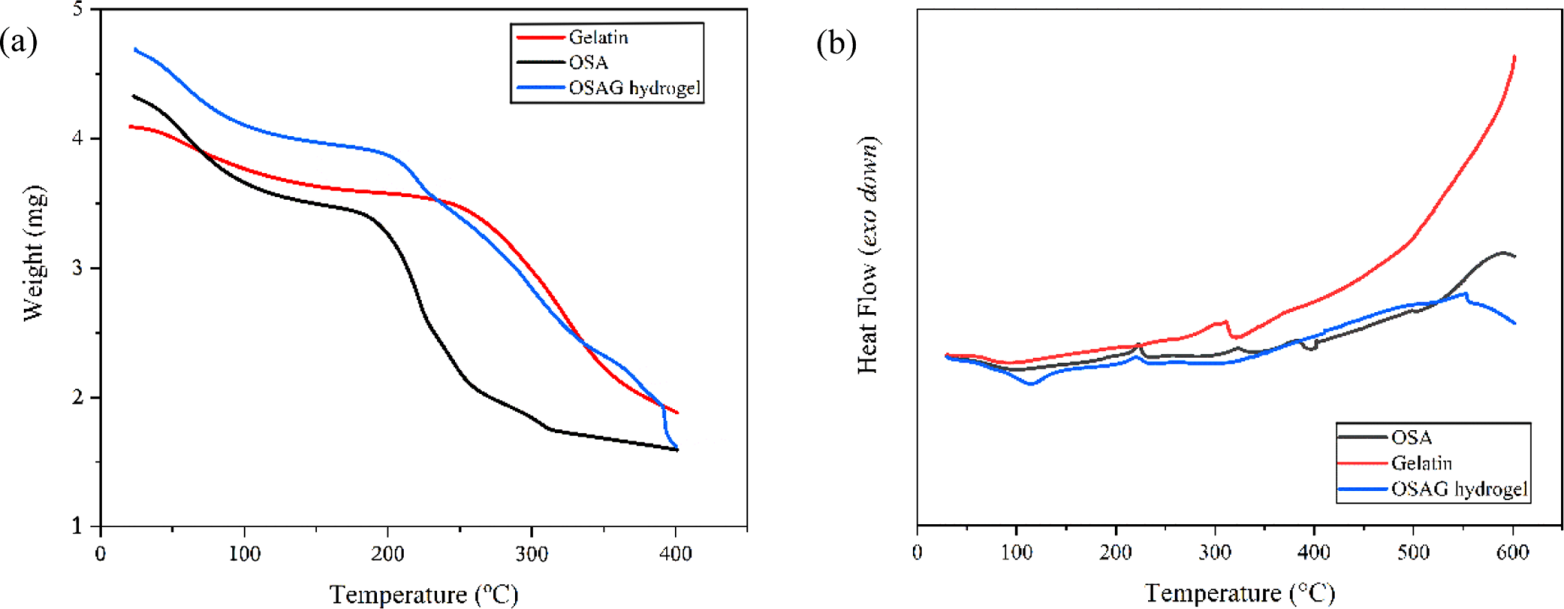
(a) TGA and (b) DSC curves of OSAG hydrogel, gelatin, and OSA.

We characterized the OSAG hydrogel, HNP, QNP and their raw materials, and OSAGH and OSAGC composite hydrogels by both TG and DSC techniques. In the TG curve of gelatin (Figure 4a, there is a two-stage decomposition profile typical of gelatins of diverse sources. The initial weight loss was up to approximately 100 °C, which corresponds to the removal of physically adsorbed water. The subsequent weight loss stage, observed at higher temperatures, was attributed to the degradation of gelatin’s proteinaceous structure, as reported past research.^44^ Whereas the OSA thermogram displayed a three-stage decomposition pattern with major weight loss between 200 and 300 °C and may be attributed to thermal degradation of functional groups.^45^ Compared with its raw materials, the TG curve of the OSAG hydrogel exhibited a multistage weight loss at higher temperatures. This could be attributed to the complex decomposition process involving various components within the hydrogel matrix. The degradation of gelatin and OSA follows distinct stages, and their combination within the hydrogel could lead to a more complex thermal profile. This complexity arises from interactions and reactions (e.g., Schiff base formation) between the polymers, possibly introducing additional stages associated with the decomposition of specific functional groups or the initiation of cross-linking reactions. These factors collectively influence the overall thermal behavior of the hydrogel.

The DSC curve (Figure 4b) of gelatin displayed two endothermic peaks associated with the denaturation and melting of gelatin, reflecting its structural changes and phase transitions. In contrast, the DSC curve of OSA revealed distinctive thermal events, including two prominent endothermic peaks at 200 and 300 °C, followed by an exothermic peak at 400 °C. As indicated by the TG analysis results, the endothermic peaks at 200 and 300 °C may be associated with specific phase transitions. The subsequent exothermic peak at 400 °C suggests the occurrence of exothermic reactions. Finally, the OSAG hydrogel DSC curve presents an exothermic peak around 100 °C, an endothermic peak around 200 °C, and another endothermic peak after 550 °C. Comparing these observations with the DSC curves of gelatin and OSA, we noted differences in the thermal behavior of the OSAG hydrogel. In contrast to gelatin, which typically exhibits distinct endothermic peaks associated with its unique structural features, and OSA, which shows specific thermal transitions due to oxidation, the hydrogel’s DSC profile reflects its complex composition and potential interactions between its components.

**Figure 5:**
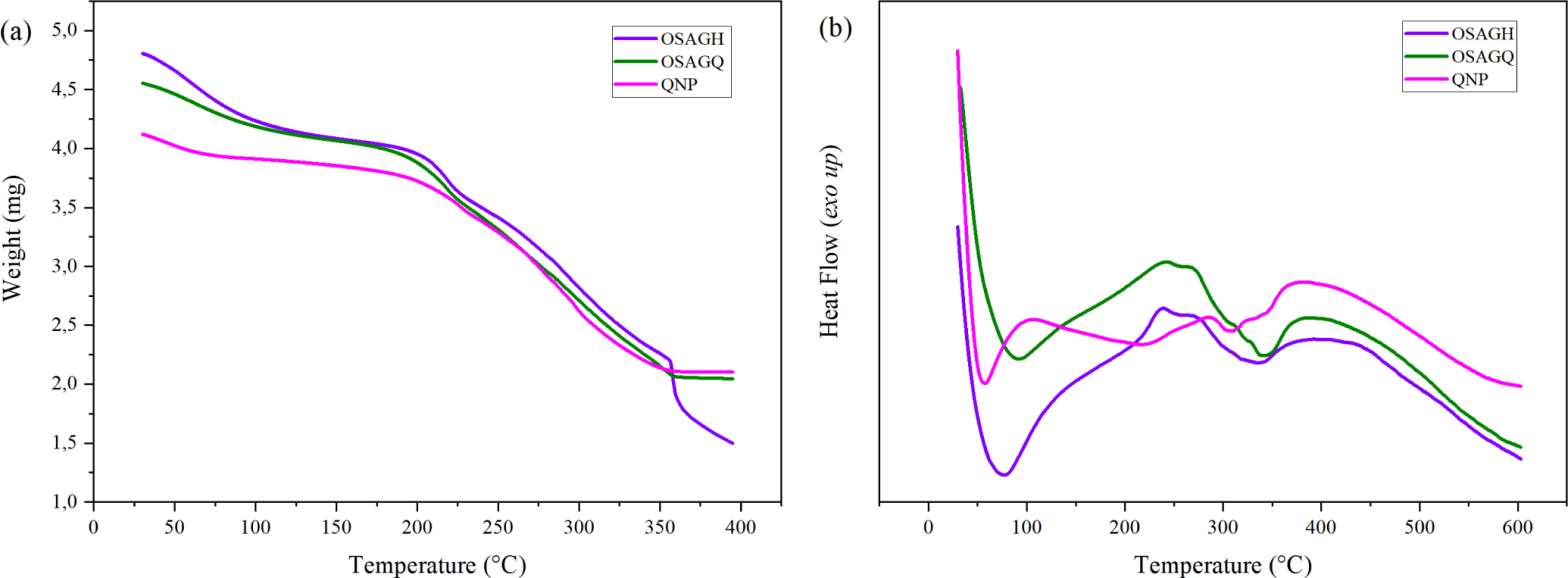
(a) TGA and (b) DSC curves of QNP, OSAGH and OSAGQ hydrogel.

Regarding the QNP and the OSAGQ hydrogel composite, it is clear that there are similarities between both weight loss patterns of quercetin-loaded nanoparticles and the nanoparticles within the hydrogel, as can be seen in Figure 5a. Upon the introduction of zein nanoparticles, changes in thermal properties were evident, indicating successful incorporation. All three materials exhibited a gradual weight loss starting before 100 °C, which can be attributed to the water evaporation.

Beyond this, a major degradation step occurred around 200–350 °C, which might correspond to the decomposition of less stable components. Interestingly, the weight loss of the OSAGH hydrogel continued after 350 °C, suggesting the breakdown of the hydrogel’s structure due to gelatin and oxidized alginate thermal decomposition.^46^ Meanwhile, the plateau-like pattern observed between 350–400 °C for the QNPs and the hydrogel loaded with them (OSAGQ) could be explained by the formation of more thermal stable structure and, thus, more stable degradation products.

The DSC curve (Figure 5b) of QNP displayed an endothermic peak at 100 °C associated with the evaporation of absorbed water as observed in the TGA, according to Campos and collaborators this endothermic event characterizes both chitosan and zein.^47^ Two exothermic peaks were observed in 200 and 300 °C, and a highest endothermic peak was observed around 400 °C, which might be related to further exothermic transitions, likely indicating the decomposition of organic components within the QNP and possibly the thermal degradation of remaining stable components or further crystallization events.

In contrast, the DSC curve of the OSAGH revealed distinctive thermal events. A prominent exothermic peak at 90 °C was observed, attributed to the evaporation of absorbed water, followed by an endothermic peak at 225 °C associated with the beginning of the decomposition of the hydrogel’s polymers, such as gelatin. Additionally, slight exothermic and endothermic peaks at 340 and 400 °C were observed, related to the disintegration of gelatin’s intermolecular side chains and further decomposition, respectively, as reported by Shehap and collaborators in their study on gelatin-based composite films.^48^ Meanwhile, OSAGQ exhibited thermal behavior similar to that of OSAGH up to 350 °C, but both OSAGQ and QNP samples showed improved thermal stability compared to OSAGH.

Considering the thermal analysis outcomes, intricate and nuanced connections exist among the different constituents of the system. Thus, the analyses provide insights into the thermal stability and decomposition behavior of the samples, which are crucial for analyzing suitability for potential applications and for understanding their performance under thermal stress. This lays the groundwork for the further refinement and optimization of this innovative drug delivery system.

#### 3.2.5 Hydrogel Rheology Measurement

**Figure 6:**
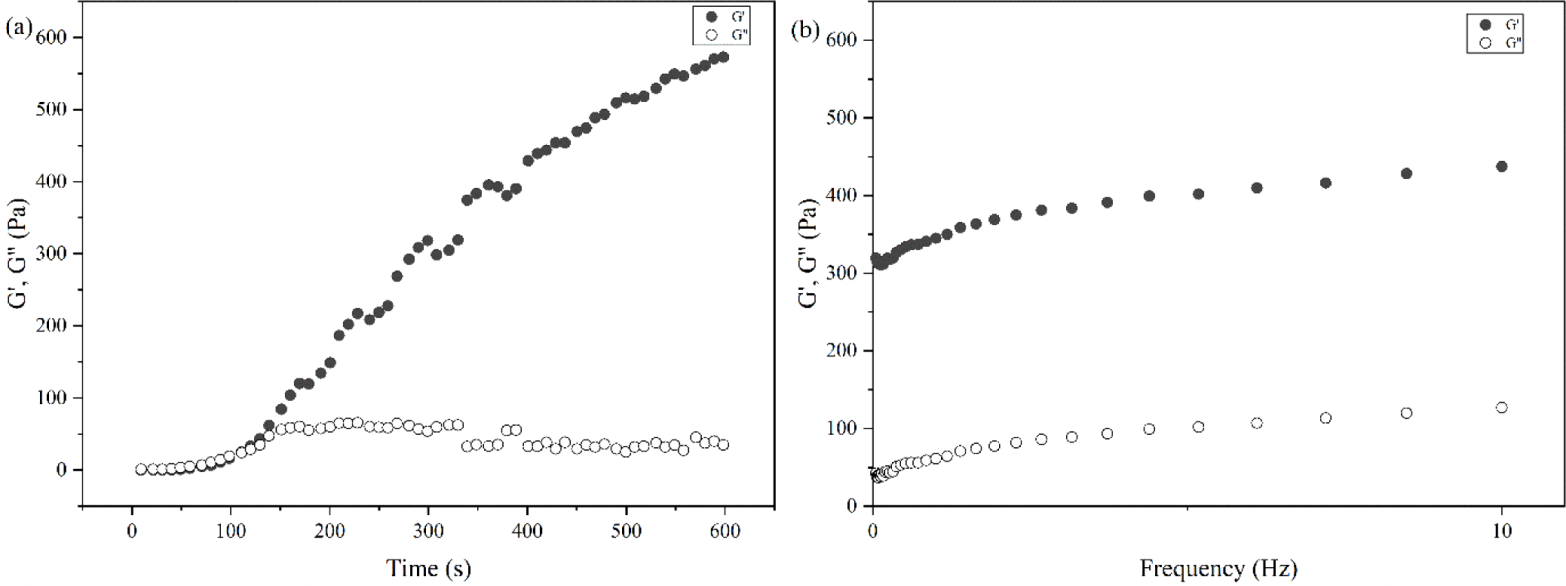
(a) Time sweep and (b) dynamic frequency sweep tests of the OSAG hydrogel.

We analyzed the rheological properties of the OSAG hydrogels using parallel plate geometry in rheological studies, as shown in Figure 6. First, a time sweep test was performed at angular frequency (1 Hz) for 10 min, shown in Figure 6a. During the process, the hydrogel storage modulus (*G*′) exceeded the loss modulus (*G*″) at 150.9 s or 2 min and 31 s, which is the critical point of the sol–gel transition and supports the gel formation results of the tube inversion test. We also conducted a dynamic frequency sweep to study the viscoelasticity and stability of the OSAG hydrogels, as shown in Figure 6b. Notably, the graph demonstrated that both *G*′ and *G*″ exhibited some frequency dependence and increased with higher frequencies. Also, within the linear viscoelastic region, the ratio of *G*″ to *G*′ was found to be less than 1, signifying the hydrogel’s ability to sustain a desirable elastic state.^49^

#### 3.2.6 Hydrogel Texture Analysis

We conducted the texture analysis of the OSAG hydrogel, without any nanoparticle, using a series of parameters shown in Table 2, including hardness, springiness, cohesiveness, adhesive force, gumminess, and chewiness.

**Table 2:** Texture Properties of the OSAG Hydrogel^a^

Sample	Hardness (N)	Springiness (mm)	Cohesiveness (N)	Adhesive Force (N)	Gumminess (N)
OSAG	1.01 ± 0.33	4.47 ± 0.24	0.87 ± 0.06	0.40 ± 0.00	0.86 ± 0.21

a Average values of the duplicate analyses. OSAG: empty hydrogel.

The hardness of the OSAG hydrogel, representing the force required to achieve a specific deformation, measured at 1.01 ± 0.33 Newtons. This indicates a low level of rigidity and resistance to deformation, which means that for the drug delivery applications and as an injectable hydrogel, less stiffness may be preferable. Conversely, a hydrogel employed in tissue engineering might necessitate increased hardness to offer mechanical support.^43^ In terms of springiness, the hydrogel exhibited 4.47 ± 0.24 mm, signifying its ability to resist being undermined by the initial compression. This resistance contributes to maintaining the integrity of the structure. The cohesiveness of the OSAG hydrogel, indicating the extent to which it resists internal rupture, was observed at 0.87 ± 0.06 Newtons. This result is higher than recently published findings of polysaccharide-based and gelatin/oxidized-starch-based hydrogels.^49,50^ Which suggests a cohesive and elastic nature, contributing to its overall stability related to the internal bonds which reflects the dense network structure of the hydrogel. The adhesive force recorded at 0.40 ± 0.00 Newtons, reflecting the ability of the hydrogel to adhere to a surface, a characteristic that could be particularly advantageous postinjection of the composite hydrogel and during the subsequent drug release phase. Gumminess, a parameter that combines hardness and cohesiveness, was determined at 0.86 ± 0.21 N, demonstrating the ability of the hydrogel to stick together and maintain its integrity. Overall, these results indicate that the hydrogel possesses favorable characteristics for drug delivery applications and as an injectable hydrogel, offers a balance between flexibility, structural integrity, and adhesiveness.

#### 3.2.7 Microstructural Analysis

**Figure 7:**
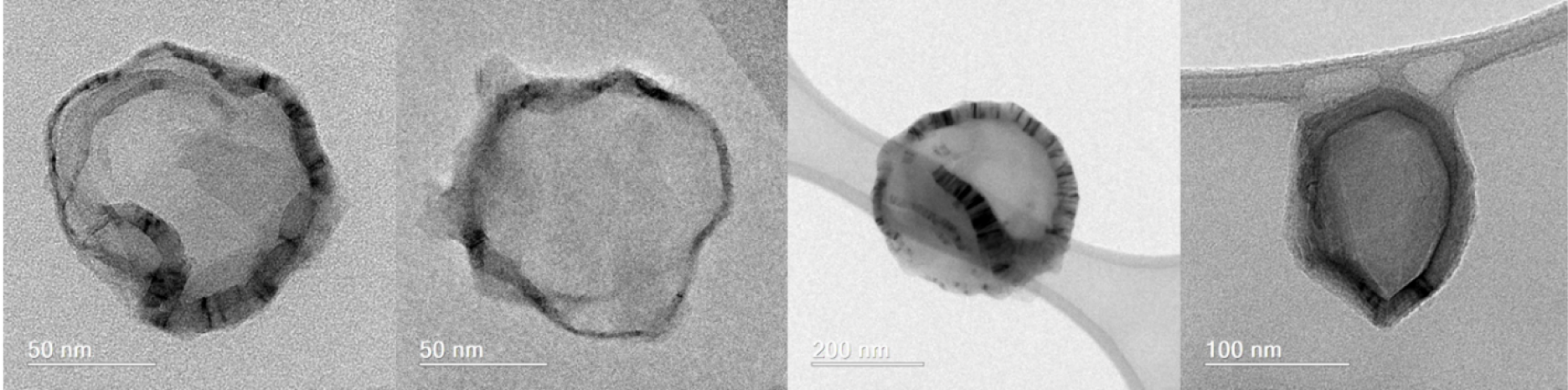
TEM images of hollow zein nanoparticles (HNP).

TEM images of HNP using a Tecnai G2–20 FEI SuperTwin 200 kV microscope are shown in Figure 7. The HNPs presented an average size between 50 to 300 nm, which is consistent with the DLS results. These particles consist of an inner lighter hollow cavity (zein protein) and a nanometric darker shell (chitosan), as described by Xu and Khan and their collaborators.^22,51^ In their work, Khan and collaborators^51^ synthesized zein particles coated with the biopolymer chitosan to evaluate the encapsulation potential of resveratrol and found morphological results very similar to those presented in the TEM images of this study.

**Figure 8:**
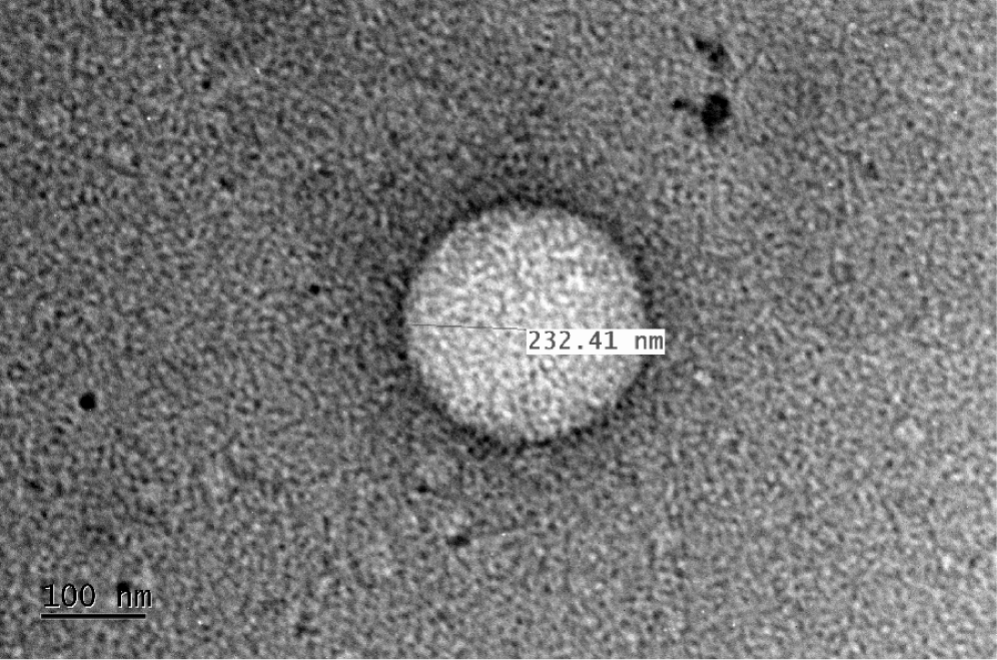
High-resolution transmission electron microscopy (HR-TEM) image showing the HNP internal cavity.

The micrographs, Figure 8, obtained using the High Resolution Transmission Electron Microscopy (HR-TEM) with a JEM-2800, 200 kV revealed a sphere-like morphology. Besides that, the image reveals a depression on the middle of the particles, more pronounced in the larger ones, indicating an internal cavity in the nanoparticles.

**Figure 9:**
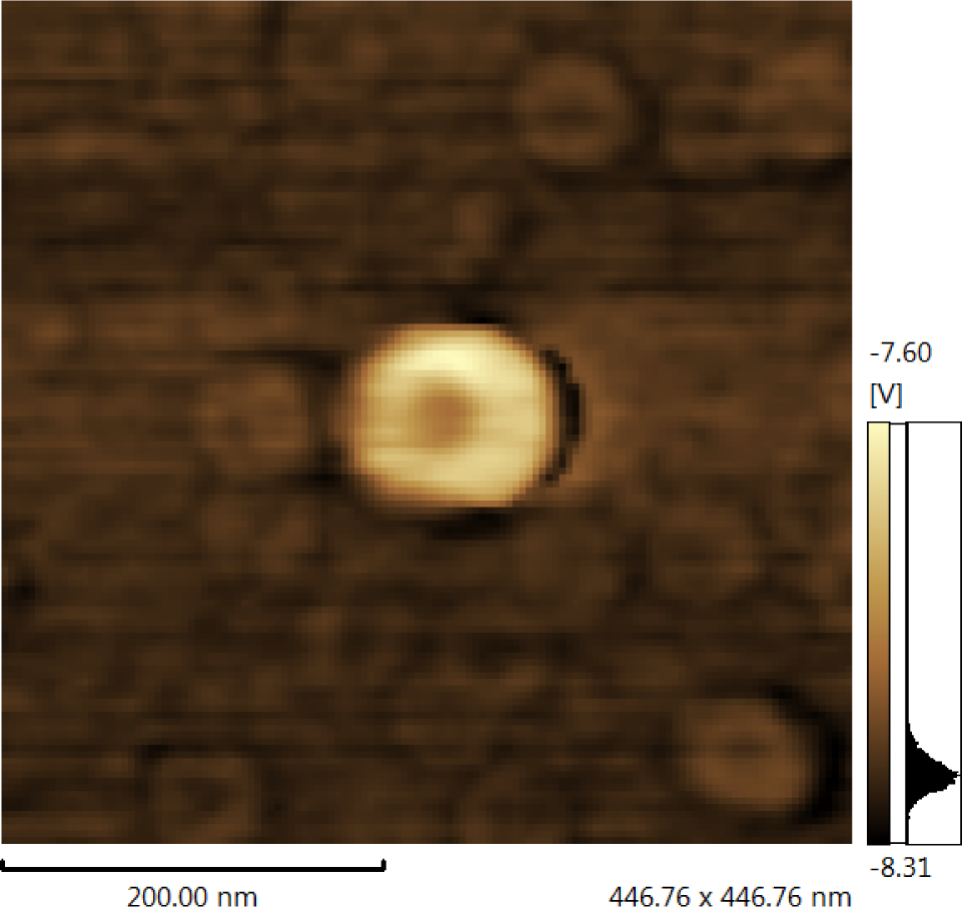
AFM images of hollow zein nanoparticle (HNP).

Finally, Figure 9 displays the AFM images. The micrographs strongly corroborate the TEM and HRTEM images. All of the hollow zein nanoparticles (NHP) display a central depression, suggesting the presence of an internal cavity, visible even in the smaller nanoparticles.

**Figure 10:**
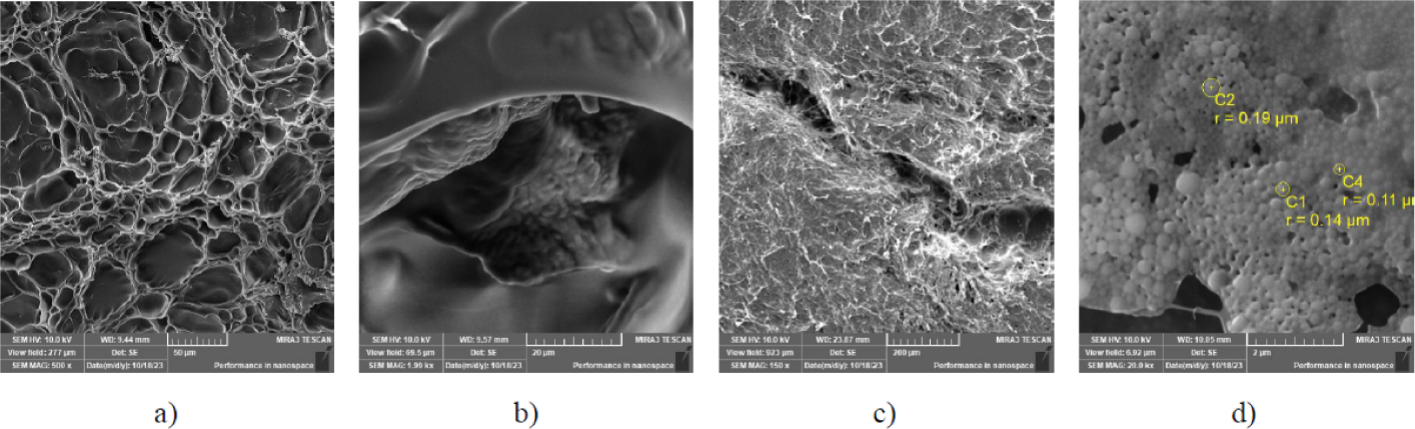
SEM images of a) OSAG (empty) hydrogel, b) hydrogel pore loaded with particles, c) self-healed OSAG hydrogel, and d) QNPs.

We used SEM analysis to determine the microstructure and morphological characteristics of the hydrogels and particles, as shown in Figure 10. In Figure 10a we can see the uneven pore distribution, creating a network rich in sites to facilitate the loading and delivery of both free-drug- and drug-loaded nanoparticles. Together with the previous characterization, results provide strong leading that enables the hydrogel to function as an effective carrier for pharmaceutical agents.

Figure 10b illustrates the hydrogel with the loaded particles within a pore. The image pictures the pore surface and an agglomerate of nanoparticles, indicating its successful loading into the hydrogel matrix.^52^Figure 10c displays the morphology of the self-healed empty hydrogel. The surface displays a unique pattern indicative of the OG hydrogel’s self-healing capability. Regions of reconnection and blending are observable diagonally, demonstrating the hydrogel’s ability to repair and regenerate its structure. Figure 10d revealed that the QNPs exhibited a spherical morphology with an average size ranging from 220 to 380 nm in diameter, which is similar to and supports those obtained when analyzed by DLS.

Additionally, the surface of the particles appeared to be smooth and was devoid of prominent surface irregularities or roughness. In summary, the SEM images offer valuable information regarding the morphological attributes of the OG hydrogel in varied states, offering insights into its structural properties, loading capacities, and ability to self-heal. These findings enhance our overall comprehension of the OG hydrogel’s potential applications, particularly in drug delivery and for various biomedical domains.

#### 3.2.8 *In Vitro* Drug Release

**Figure 11:**
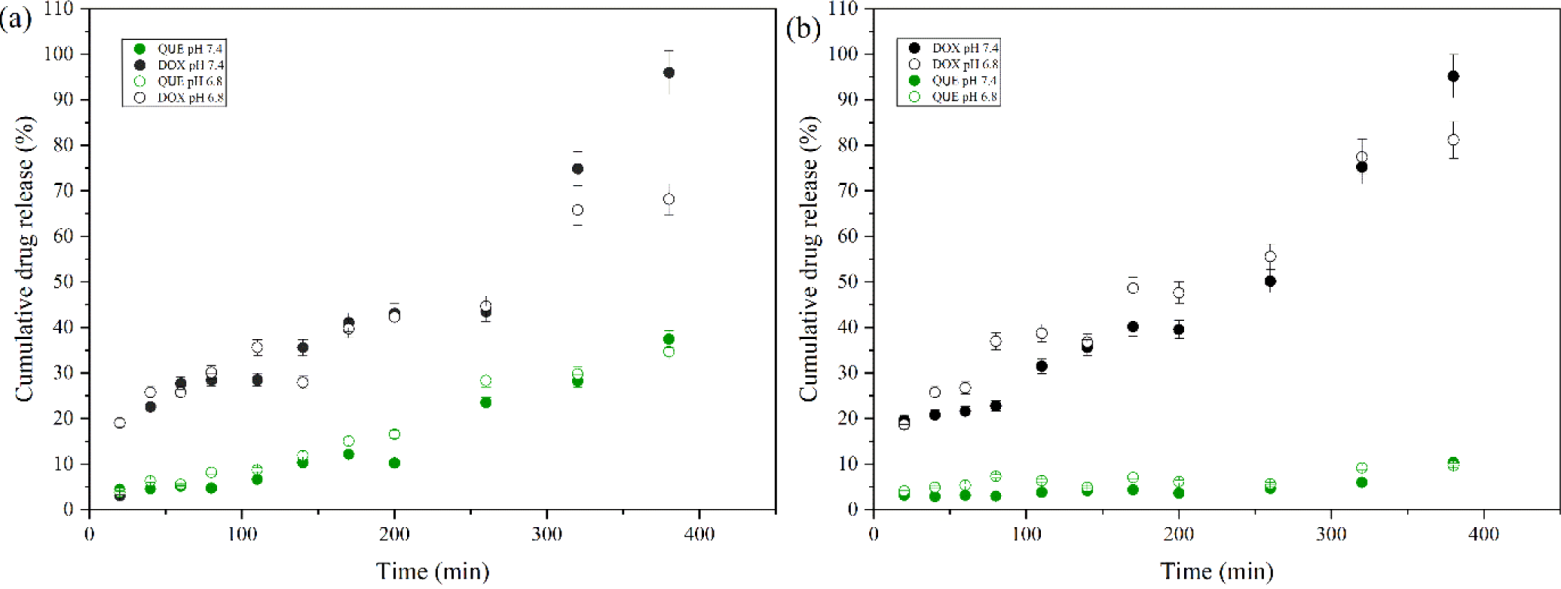
Comparative drug release profiles of QUE and DOX from hydrogel composites at pH 7.4 and pH 6.8: a) Individually and b) combined, respectively.

In our pursuit to provide a way to enhance chemotherapy efficacy while reducing systemic toxicity,^6^ the dual drug release behavior of the hydrogel in environments of pH 7.4 and pH 6.8, representing physiological and tumorous conditions, respectively, was assessed. The individual release of quercetin exhibited a sustained and consistent profile at pH 7.4, with a maximum cumulative release of 37.5% over 6.5 h, as shown in Figure 11a. At pH 6.8, the quercetin release profile displayed similar characteristics with the cumulative release being slightly lower at 34.7%. This finding could be attributed mainly to the less pronounced swelling behavior of the hydrogel at pH 6.8 due to lower ionization of carboxyl groups, leading to slightly reduced water uptake and, consequently, slightly lower diffusion rates. Additionally, since QNP is coated with chitosan, at pH 6.8, chitosan is more protonated, increasing its solubility and swelling capacity, which could slightly increase the release rate of quercetin from the nanoparticles. However, the overall release rate remains consistent due to the hydrogel’s restricted swelling at this pH. The drug release of doxorubicin from the hydrogel matrix showed similar release profiles at both pH 7.4 and 6.8 with distinct cumulative release amount. At pH 7.4, the cumulative release reached 95.9%, while at pH 6.8, the release was comparatively lower, reaching 68.2%. Notably, the release behavior at pH 6.8 indicated a more gradual release pattern over the 6.5-h analysis period, contrasting with the release observed at pH 7.4. The observed differences attributed to swelling patterns, may have influenced the rate of the doxorubicin release, especially at pH 6.8. Since at pH 6.8 the reduced swelling means smaller diffusion channels, which hinders the release of DOX. Besides that, DOX has a p*K*_a_ 8.25, so at pH 6.8, the protonated doxorubicin may interact more strongly with negatively charged groups within the hydrogel, reducing its diffusion rate.^53^ Finally, quercetin had to overcome more barriers for release, involving both the particle and the hydrogel matrix, whereas doxorubicin was simply dispersed within the hydrogel polymeric matrix.

In the combined drug release (Figure 11b), only quercetin exhibited a distinct release profile compared to its individual release, resulting in a slightly lower release amount. Meanwhile, the cumulative release of doxorubicin from the hydrogel was higher at pH 7.4 compared with pH 6.8, consistent with the individual release experiment. The reduced swelling at pH 6.8 restricted the diffusion of both drugs, leading to lower cumulative release percentages.

Simultaneous loading also introduced complexities that contribute to this phenomenon. For instance, at pH 7.4, doxorubicin is less protonated, resulting in fewer interactions with the hydrogel matrix and QNP, and thus higher release. Additionally, the chitosan coating on zein nanoparticles imparts a positive surface charge, facilitating electrostatic interactions between these nanocarriers and the negatively charged groups in the hydrogel matrix as observed by Campos and collaborators.^47^ These interactions could lead to a more controlled release in smaller proportions compared to the release of doxorubicin.

The Peppas-Sahlin mathematical model was best fitted (r^2^ > 0.98) for individual quercetin release at pH 6.8 among the five mathematical models considered, suggesting a complex release mechanism beyond simple diffusion. In contrast, under all other release conditions—both individual and combined—the zero-order model exhibited the best-fitted profile, indicating a constant-rate release mechanism independent of drug concentration. Thus, the quercetin release profile described by a different model than those under the other release conditions is consistent with the differences observed in the release profiles previously depicted.

### 3.3 *In Vitro* Cytotoxicity

The results of the cytotoxicity evaluated in human tumor cell line MCF-7 are expressed in Table 3 as the concentration that inhibits cell growth by 50% (IC_50_), for each treatment within 72 h.

**Table 3:** IC50 Values for MCF-7 Cell Growth Inhibition Following 72 h Treatments with the Samples, Determined by the MTT Assay

Samples	IC_50_ (μg/mL)
DOX	0.331 ± 0.013
QUE	>6
QNP	>6
OSAGC	0.016 ± 0.014
OSAGX	0.071 ± 0.039
OSAGQ	2.07 ± 0.24

Data obtained from three independent experiments performed in triplicate or quadruplicate and presented as a concentration that inhibits cell growth by 50% (IC50) ± SEM, obtained by nonlinear regression. DOX: doxorubicin (alone); QUE: quercetin (alone); QNP: quercetin-loaded nanoparticles; OSAGQ: hydrogel + QNP; OSAGX: hydrogel + DOX; and OSAGC: hydrogel + DOX + QNP.

QUE and QNP were the only samples that did not significantly reduce the cell viability. Remarkably, when compared to doxorubicin alone, the OSAGX formulation potentiated cytotoxic action by 4.66 times, while the primary formulation of OSAGC enhanced it by 20.7 times. When comparing OSAGX and OSAGC, the addition of QNP additionally increased the cytotoxicity by 4.4 times.

In a study by Hassan and collaborators,^19^ QUE was found to enhance the cytotoxic activity of doxorubicin on pancreatic adenocarcinoma cell line AsPC-1 and human hepatocellular carcinoma cell line HepG2 through synergistic apoptotic effects, inhibition of HIF-1α, and MDR1 activity, which supports our findings. This is also consistent with previous *in vitro* research by Zhang and collaborators^20^ where QUE was shown to enhance the cytotoxic activity of doxorubicin on breast cancer cells, highlighting the potential of QUE as an adjuvant in cancer treatment.

These findings suggest that this innovative system enhances the toxic effect of DOX, offering a promising approach to improving cancer treatment efficacy through dual-drug synergistic effects. Consequently, the formulated system represents a promising platform for the combined delivery of hydrophobic and hydrophilic substances, such as quercetin and doxorubicin, respectively, thereby increasing the applicability of this type of hydrogel.

## 4 Conclusions

Our investigation successfully incorporated doxorubicin into the hydrogel matrix and loaded quercetin onto chitosan-coated zein particles, establishing a composite system. The spectroscopic results, in conjunction with thermal analysis, collectively provided comprehensive insight into the complexity of interactions between constituents in the hydrogel composite. The hydrogels displayed the anticipated self-healing property, attributed to Schiff base bonds, and demonstrated injectability. They also revealed a porous structure, illustrating the hydrogel’s capacity to load both doxorubicin- and quercetin-loaded zein particles. Exposure of our hydrogel composite formulation to MCF-7 breast cancer cells demonstrated an enhanced toxic effect compared with doxorubicin alone. Thus, this innovative system introduces a potent therapeutic outcome in combination therapy.

## Supporting Information Available

Quercetin and Doxorubicin standard curves; FTIR spectra hydrogel, particles and their raw; ^1^H NMR spectra of hydrogel raw material and ^1^H–^13^C HSQC spectrum of hydrogel raw (PDF)

## Abbreviations

QUE: quercetin
DOX: doxorubicin
HNP: empty nanoparticles
QNP: nanoparticles + quercetin
OSAG: empty hydrogel
OSAGH: hydrogel + HNP
OSAGQ: hydrogel + QNP
OSAGX: hydrogel + DOX
OSAGC: hydrogel + DOX + QNP
