# Supplementary material for: Gelatin-Oxidized Alginate and Chitosan-Coated Zein Nanoparticle Hydrogel Composite to Enhance Breast Cancer Cytotoxicity in Dual-Drug Delivery
Source: ACS Omega. 2024 Oct 31;9(45):45190–202. doi: 10.1021/acsomega.4c06404 (PMC11561619; doi:10.1021/acsomega.4c06404)
Supplement: Supplementary file 1 — ao4c06404_si_001.pdf [file ao4c06404_si_001.pdf]

Gelatin-Oxidized Alginate and Chitosan-Coated Zein Nanoparticles Hydrogel Composite to Enhance Breast Cancer Cytotoxicity in Dual-Drug Delivery

Sanierlly da Paz do Nascimento[1], Ramon Ramos Marques de Souza[2], Marianna Vieira Sobral[2,5], Francisco Humberto Xavier-Junior[2,5], Marcus Vinícius Santos da Silva[3], Marcelo Machado Viana[4], Fausthon Fred da Silva[1,6], Michael J. Serpe[7], Antonia L. de Souza[1,6*]

[1]Postgraduate Program in Chemistry, Universidade Federal da Paraíba, João Pessoa, 58051-900 PB, Brazil

[2]Postgraduate Program in Natural and Synthetic Bioactive Products, Universidade Federal da Paraíba, João Pessoa, 58051-900 PB, Brazil

[3]Institute of Physics, Federal University of Bahia, Salvador, 40170-290 BA, Brazil

[4]CTNano, Universidade Federal de Minas Gerais, Belo Horizonte, 31270-901 MG, Brazil

[5]Department of Pharmaceutical Sciences, Universidade Federal da Paraíba, João Pessoa, 58051-900 PB, Brazil

[6]Department of Chemistry, Universidade Federal da Paraíba, João Pessoa, 58051-900 PB, Brazil

[7]Department of Chemistry, University of Alberta, Edmonton, T6G 2R3 Alberta, Canada

*Corresponding author: Antonia Lucia de Souza. Current e-mail addresses:

antonia_lucia@yahoo.com.br

Supporting Information

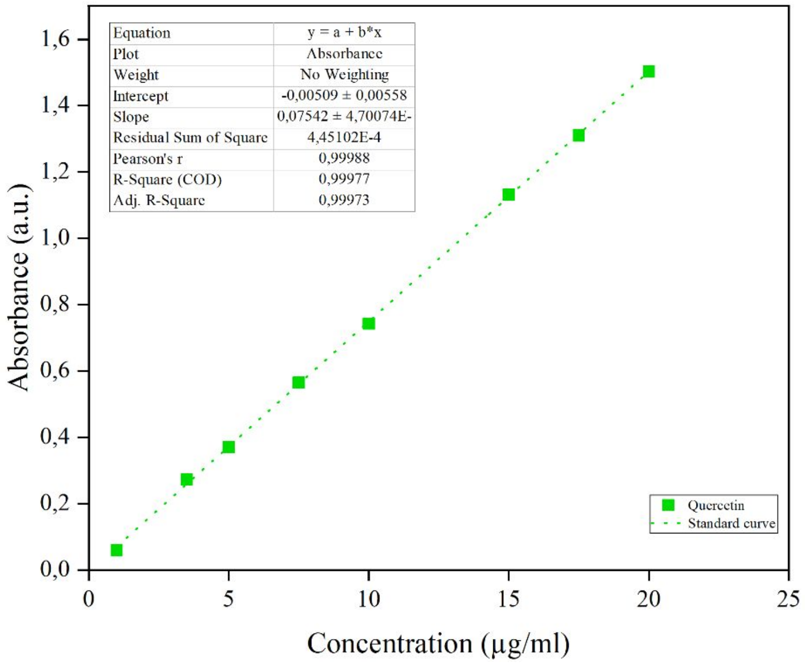

Figure S1. Quercetin calibration curve (λ = 373 nm) for loading capacity and efficiency

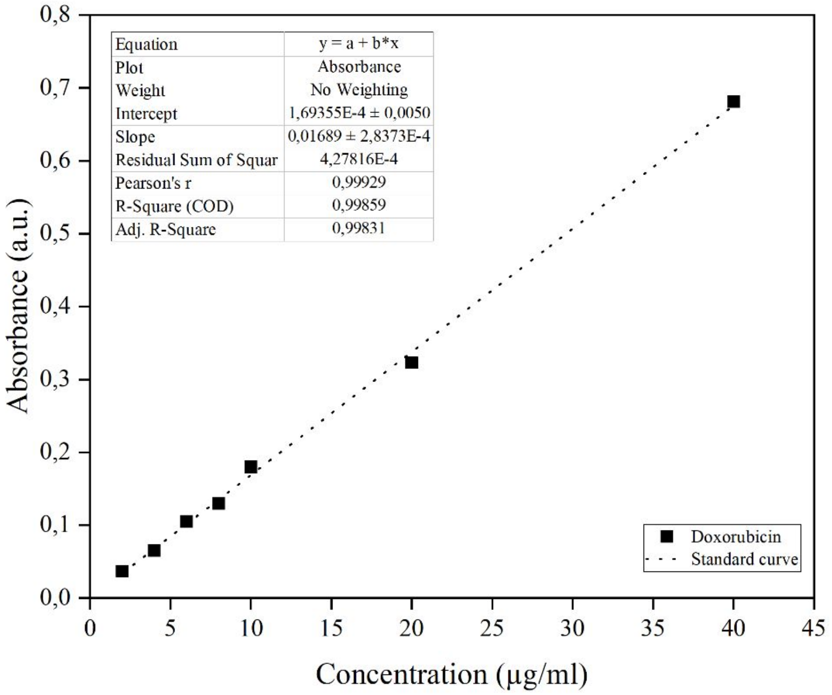

and release.

Figure S2. Doxorubicin calibration curve (λ = 478 nm) for drug release experiments.

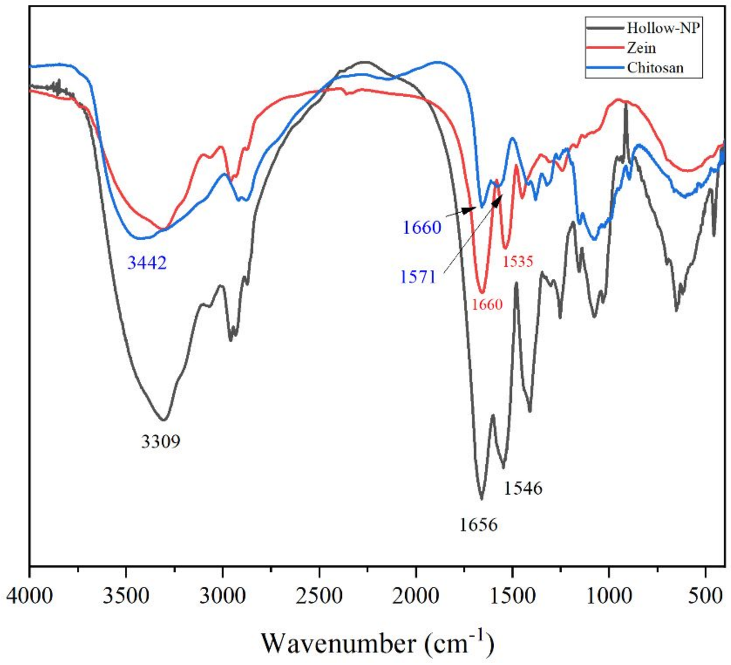

Figure S3. FTIR spectra of hollow chitosan-coated zein nanoparticles (NP) and its raw materials: Zein and chitosan.

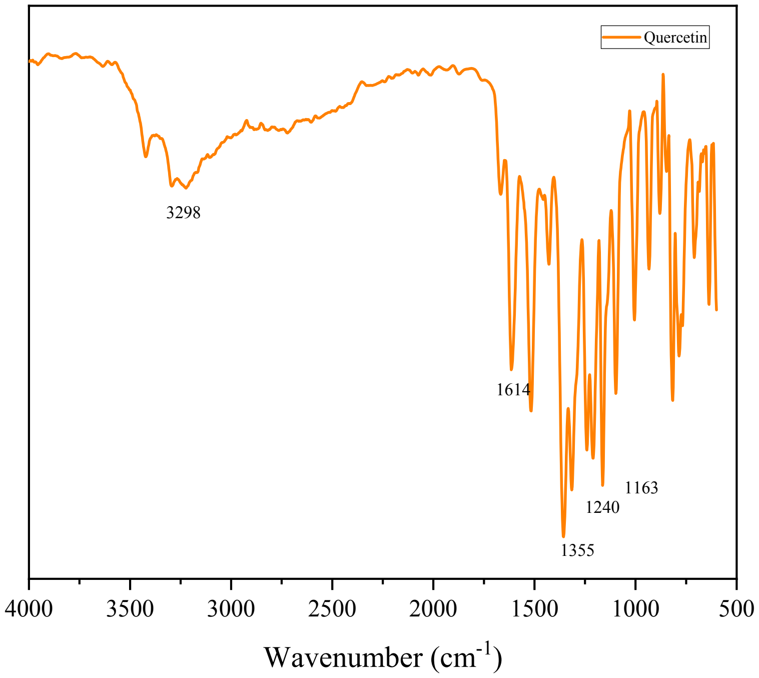

Figure S4. FTIR spectrum of quercetin.

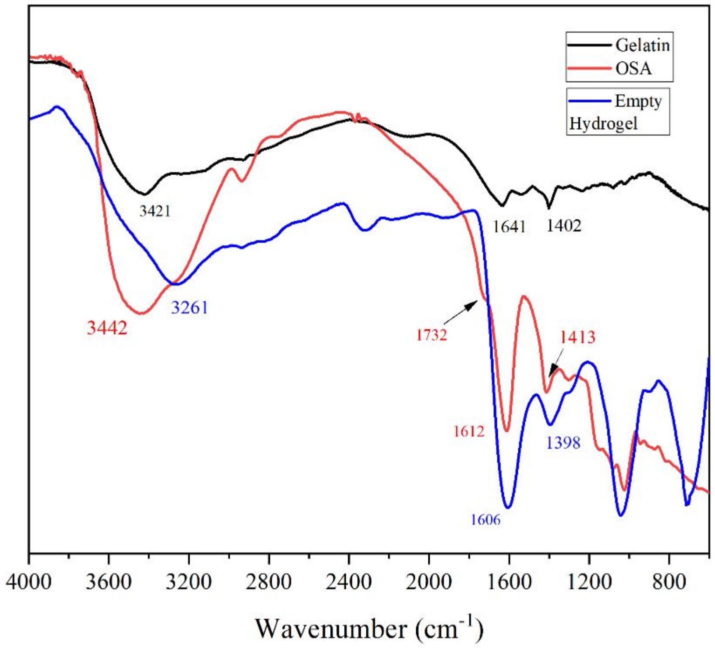

Figure S5. FTIR spectra of the empty Oxidized Alginate-Gelatin (OG) hydrogel and its raw materials: gelatin and oxidized alginate (OSA).

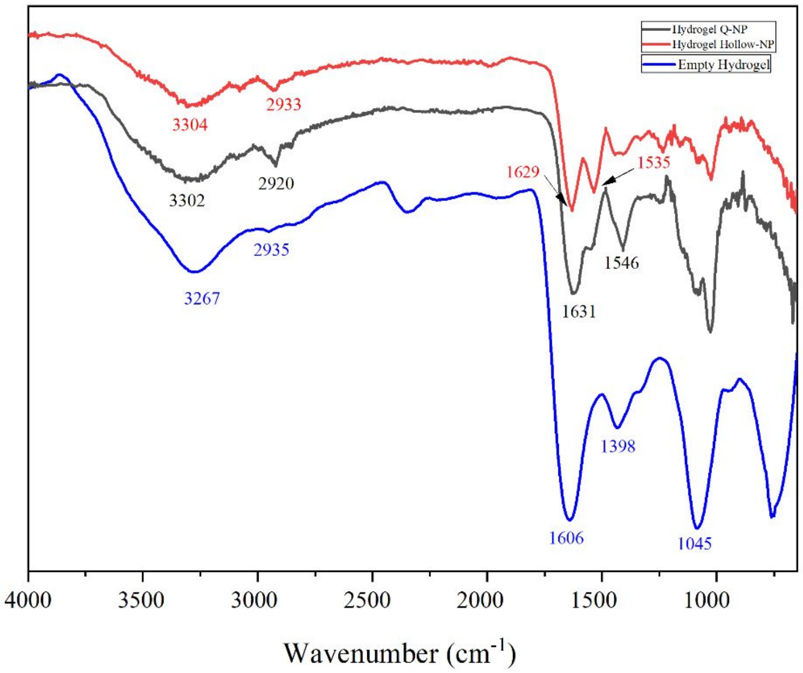

Figure S6. FTIR spectra of OG hydrogels: empty, loaded with hollow-nanoparticle (Hollow-NP), and loaded with quercetin-loaded nanoparticle (Q-NP).

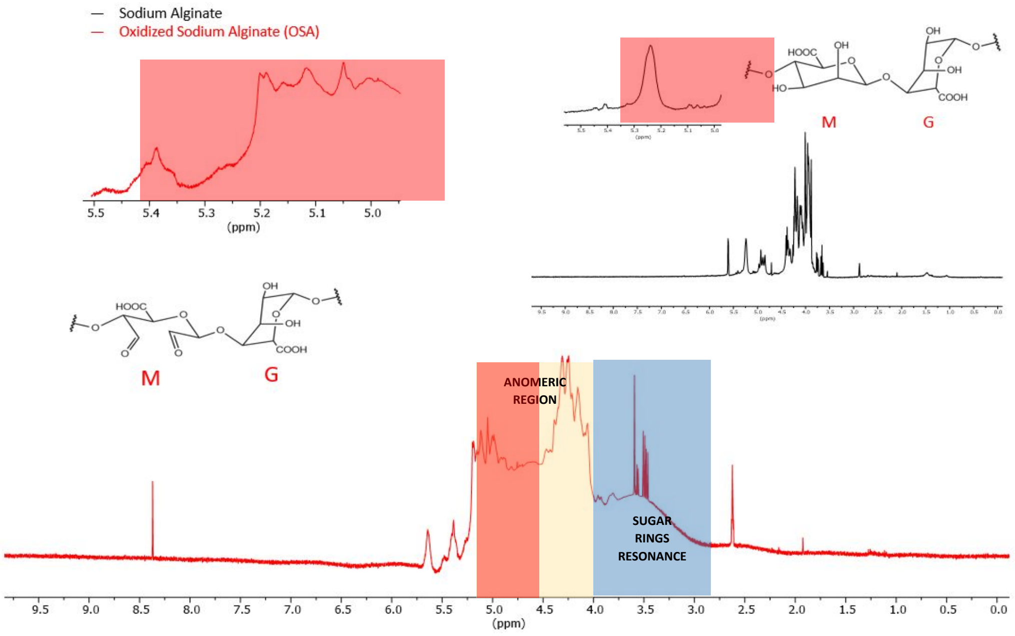

Figure S7. Sodium alginate and Oxidized Sodium Alginate ^{1}H-Nuclear magnetic resonance (^{1}H-NMR, D2O) spectra.

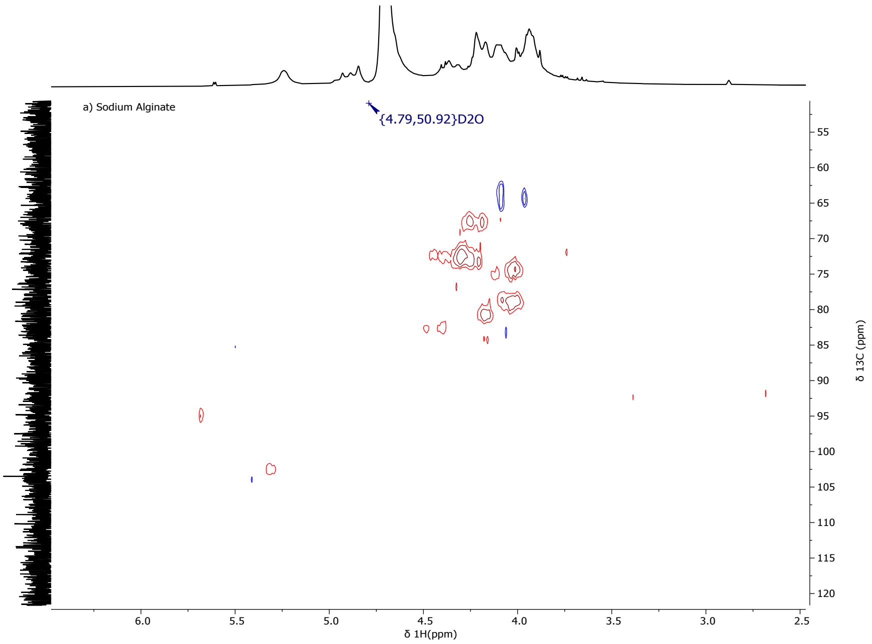

Figure S8. ^{1}H–^{13}C HSQC spectra of sodium alginate (SA) (a) and oxidized sodium alginate (OSA) (b) in D2O/DMSO-d6 at RT (400 MHz NMR spectrometer).

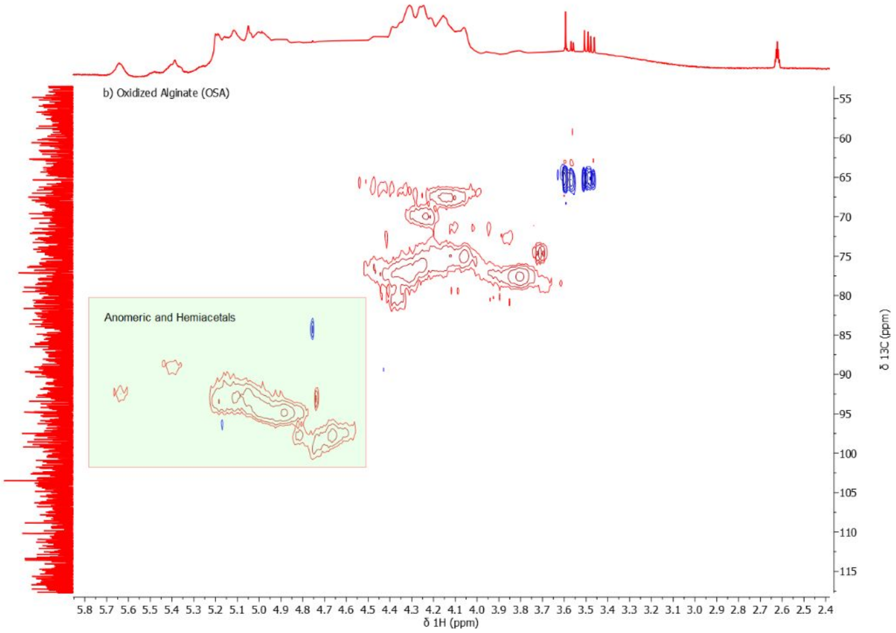

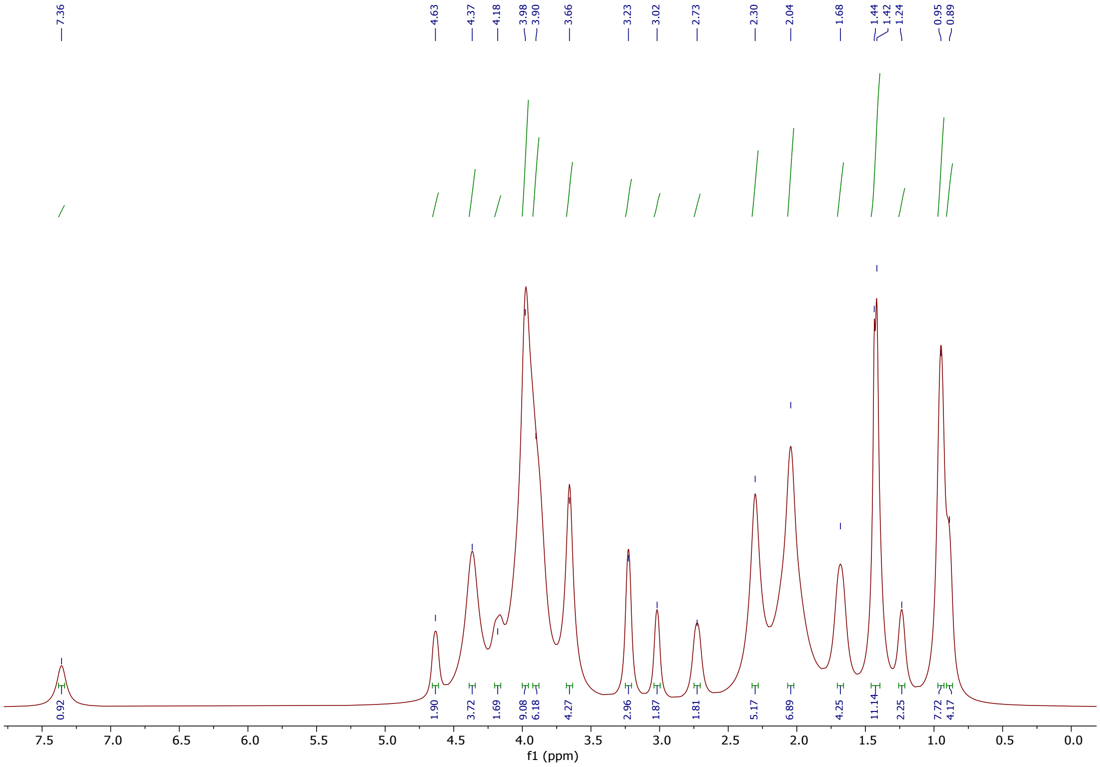

Figure S9. Gelatin ^{1}H-Nuclear magnetic resonance (^{1}H-NMR) spectrum in D2O at RT in a 400 MHz NMR spectrometer.
